# Terahertz Time-Domain Spectroscopy as a Defect Fingerprinting Tool for Halide Perovskite Solar Cells: Toward a Universal Framework

**DOI:** 10.3390/nano16171072

**Published:** 2026-08-28

**Authors:** Inhee Maeng, Young Mi Lee, Jinwoo Park, Seung Jae Oh, Min-Cherl Jung

**Affiliations:** 1 YUHS-KRIBB Medical Convergence Research Institute, College of Medicine, Yonsei University, Seoul 03722, Republic of Korea; issac@yuhs.ac; 2 Beamline Division, Pohang Accelerator Laboratory, Pohang University of Science and Technology, Pohang 37673, Republic of Korea; yiymi35@postech.ac.kr; 3 Department of Physics, University of Seoul, Seoul 02504, Republic of Korea; reduck96@physics.uos.ac.kr; 4 Global Institute of Future Technology, Shanghai Jiao Tong University, Shanghai 200240, China

**Keywords:** terahertz time-domain spectroscopy, organic halide perovskite, defect fingerprinting, grain boundary, sequential vacuum evaporation, CH_3_NH_2_ molecular defect, phonon modes, MAPbI_3_, MAPbBr_3_, CsPbI_3_

## Abstract

Organic–inorganic hybrid perovskites (OHPs) deliver certified single-junction power conversion efficiencies (PCEs) of up to 27.3% and National Laboratory of the Rockies (NLR)-certified perovskite–silicon tandem values of 34.85%, yet a substantial gap with the Shockley–Queisser (S–Q) limit persists. Grain-boundary (GB) defects are one principal contributor to this gap, driving non-radiative recombination, ion migration, and degradation alongside bulk, interfacial, contact-related, phase-related, and environmental loss channels. Rational passivation demands a non-contact tool capable of identifying and quantifying specific defect species in device-relevant thin films, a capability that conventional probes deliver only in part. This overview assesses the extent to which terahertz time-domain spectroscopy (THz-TDS, 0.2–2.5 THz) fulfills this role. Across five OHP compositions—MAPbI_3_, MAPbBr_3_, FAPbI_3_, and FAPb(Br,I)_3_ fabricated by sequential vacuum evaporation (SVE), together with solution-processed *γ*-CsPbI_3_—the THz spectral window captures both intrinsic phonon modes and GB-localized molecular defect vibrations, enabling species-resolved characterization at room temperature. Notably, the oscillator strength of the SVE-specific 1.58 THz absorption in MAPbI_3_ scales linearly with XPS-quantified CH_3_NH_2_ defect concentration, establishing a calibrated, contact-free proxy for defect concentration rather than an absolute defect count; the observable is the defect-induced perturbation of the Pb–X lattice, not the defect population itself. Building on these findings, we propose a three-pillar framework for THz-guided defect engineering: (I) quantitative defect measurement via oscillator-strength analysis, (II) material-specific fingerprint identification from a systematically constructed THz library, and (III) fingerprint-guided defect elimination with real-time feedback—together defining a closed-loop quality-control cycle that connects spectroscopic diagnosis to passivation strategy and, ultimately, to enhanced solar cell efficiency. Throughout, we distinguish capabilities demonstrated to date from extensions that remain proposals, and we define the measurement requirements needed before the framework can be transferred to inline manufacturing control.

## 1. Introduction

Photovoltaic research today spans several distinct material families. Crystalline silicon remains the industrial standard; binary oxide ceramics such as TiO_2_, ZnO, Al_2_O_3_, SiO_2_, CeO_2_, Fe_2_O_3_, and WO_3_ serve as electron-transport layers, antireflective coatings, passivation layers, and buffer layers across silicon, dye-sensitized, thin-film, and perovskite architectures [1]; and luminescent metal oxides act as spectral modifiers that reshape the incident solar spectrum to better match absorber bandgaps [2]. Chalcogenide thin films (CdTe, CIGS) and organic photovoltaics occupy further niches defined by cost, flexibility, and form factor.

Organic–inorganic hybrid perovskites (OHPs) of the ABX_3_ crystal family (A = CH_3_NH_3_^+^ [MA], CH(NH_2_)_2_^+^ [FA], Cs^+^; B = Pb^2+^; X = I^−^, Br^−^) have emerged as one of the most disruptive developments in photovoltaics over the past decade [3]. From a PCE of 3.8% in 2009, certified single-junction OHP solar cells have now reached 27.3%, as listed in the National Laboratory of the Rockies (NLR) Best Research-Cell Efficiency Chart [4,5], approaching silicon in single-junction configuration and surpassing it in tandem. LONGi Green Energy reported a perovskite–silicon two-terminal tandem efficiency of 34.85% in April 2025, as certified by the National Renewable Energy Laboratory (NREL) [6], exceeding the Shockley–Queisser (S–Q) limit for single-junction devices [7] and underscoring the transformative commercial potential of OHP technology; a higher value of 35.5%, certified by the European Solar Test Installation (ESTI), was announced in July 2026 but has not yet entered the NLR tabulation and is quoted here only for context. These advances rest on the exceptional intrinsic properties of OHPs: tunable bandgaps of 1.1–3.0 eV by halide substitution, absorption coefficients exceeding 10^4^ cm^−1^, long carrier diffusion lengths (>1 μm in thin films, >175 μm in single crystals), and scalable deposition by solution and vacuum methods [3,8].

Despite these achievements, the gap between laboratory-record efficiencies and the S–Q theoretical limit (~30.5% for the ~1.55 eV bandgap of MAPbI_3_; the absolute S–Q maximum of ~33.7% occurs near 1.34 eV) remains substantial, and long-term operational stability under heat, humidity, and illumination continues to impede commercialization. These shortcomings are not attributable to any single mechanism. Efficiency and stability losses in OHP devices originate from bulk point defects, grain boundaries (GBs), absorber–transport-layer interfaces and contacts, phase instability, and environmental degradation, and their relative weight varies with composition, architecture, and operating condition [9,10]. Within this set, GB defects in polycrystalline thin films occupy a particularly influential position, and they are the focus of the present review. GB defects in OHP act as deep-level non-radiative recombination centers, reducing the open-circuit voltage (V_OC_) and fill factor (FF) below their radiative limits [9]. Ion migration through GB defect pathways drives phase segregation, halide redistribution, and irreversible degradation under operating conditions [11,12]. Consequently, GB defect passivation has emerged as a central engineering challenge for achieving simultaneously high efficiency and stability in OHP photovoltaics. Recent strategies—including ammonium-salt treatments, sulfonium-based passivators, and ultra-thin polymer interlayers—have extended device lifetimes to thousands of hours under continuous illumination [13,14]. The relevance of GB defect control extends beyond photovoltaics: the same ionic mobility and defect chemistry underpin halide perovskite memristors, in which Cs_3_Bi_2_I_6_Br_3_ devices have been shown to reproduce spike-timing-dependent plasticity for neuromorphic computation [15].

Maximizing the impact of defect passivation strategies critically depends on the ability to identify, classify, and quantify the specific defect species present in each OHP composition and fabrication method. Yet, this remains a formidable analytical challenge. In each comparison below, the limitation is one of practical accessibility rather than absolute incapability. Conventional structural probes—X-ray diffraction (XRD) and scanning electron microscopy (SEM)—reveal grain morphology and crystallographic phase with high reliability but are not, in their standard implementations, sensitive to the molecular-level chemical states of GB defects. Photoluminescence (PL) spectroscopy provides defect-density information through non-radiative recombination rates; advanced implementations such as hyperspectral and time-resolved PL imaging offer considerable spatial and kinetic resolution, but the signal still convolutes multiple recombination pathways and does not by itself distinguish specific defect chemical species [16]. X-ray photoelectron spectroscopy (XPS) directly identifies defect chemical states with high sensitivity but is surface-limited (~10 nm probing depth), requires high-vacuum sample transfer, and cannot be applied non-destructively to device-integrated films [16]. Impedance spectroscopy accesses trap densities through device-level measurements but requires electrical contacts, and the extraction of defect densities from capacitance data is model-dependent and can be ambiguous [17]. Deep-level transient spectroscopy and *in situ/operando* synchrotron methods extend these capabilities considerably but require either contacts or facility-scale infrastructure [18]. Consequently, comprehensive, non-contact, direct characterization of molecular defect species in OHP GB regions—the very defects that limit efficiency—remains an unmet need.

Terahertz time-domain spectroscopy (THz-TDS) operates in the 0.1–3.0 THz range (0.4–12.4 meV), a spectral window that directly overlaps with: (i) low-energy phonon vibration modes of OHP lattices (Pb–X cage vibrations, organic-cation librations), (ii) molecular vibrations of defect species at GBs, and (iii) intraband free-carrier dynamics. As a contact-free, non-destructive probe operating at room temperature under ambient conditions, THz-TDS is well suited to direct, species-resolved defect characterization. Early optical pump-terahertz probe (OPTP) studies by Ponseca et al. [19] confirmed ultrafast, balanced free carriers in MAPbI_3_ with a sum mobility (μ_e_ + μ_h_ ≈ 25 cm^2^ V^−1^ s^−1^), establishing THz-TDS as a benchmark for OHP carrier quality. Subsequent work revealed phase-dependent phonon mode transformations [20], optical phonon–carrier coupling [21], and electron–phonon interactions [22] using THz spectroscopy. Near-field THz nanoimaging recently demonstrated GB-resolved trap mapping with sub-20 nm spatial resolution [23]. Together, these studies establish THz-TDS as a powerful yet underexploited probe of carriers, phonons, and traps in OHPs. Species-specific chemical identification, by contrast, has been demonstrated only for particular material–process combinations rather than as a general capability, and the present review preserves that distinction throughout.

A previous review by the present authors surveyed THz-wave absorption in OHP materials from the perspective of THz sensor development [24]. The present work differs in aim and organization: rather than treating THz absorption as a device-functional property, it treats the THz spectrum as a diagnostic observable for defect chemistry, introduces an explicit separation between demonstrated and proposed capability, and formulates the resulting workflow as a three-pillar engineering framework with defined validation requirements.

In this overview, we present a comprehensive account of THz defect fingerprints in OHP samples, most of which were fabricated by sequential vacuum evaporation (SVE)—a scalable, substrate-universal vacuum deposition method. The library comprises polycrystalline thin films of MAPbI_3_ (solution-processed and SVE), MAPbBr_3_ (SVE), FAPbI_3_ (SVE, on flexible substrates), FAPb(Br,I)_3_ (SVE), and *γ*-CsPbI_3_ (solution-processed), together with single-crystal data for FAPbI_3_. No measurements on complete, contacted solar cells are included; the implications of this omission for device translation are treated in Section 6. By systematically mapping THz fingerprints across these compositions, we construct a material-specific THz defect library and show how it enables a three-pillar engineering cycle that closes the loop between defect identification and elimination.

## 2. Defects in OHP Thin Films: Classification, Origin, and Impact

Defects in polycrystalline OHP thin films originate from multiple sources and manifest in distinct chemical and structural forms. Point defects—including halide vacancies (V_I_, V_Br_), B-site vacancies (V_Pb_), A-site vacancies (V_MA_), interstitials (I_i_, Pb_i_), and antisites—form within grains with formation energies governed by chemical potential [25,26]. Among these, iodine interstitials and MA-on-Pb antisites create deep trap levels (>0.5 eV from band edges) that dominate non-radiative recombination in MAPbI_3_. Grain boundaries concentrate these point defects due to under-coordinated Pb^2+^ and X^−^ sites at the lattice termination, making GBs the primary recombination-active regions in polycrystalline films [27].

The fabrication method profoundly determines GB defect chemistry. Solution-processed OHP films form GBs during solvent evaporation with residual solvent molecules and incomplete crystallization creating point-defect clusters. Vacuum-deposited films formed by SVE (sequential deposition of MAI and PbI_2_ in separate steps) create smaller, denser grain structures with higher GB area density. Critically, incomplete perovskite reaction under vacuum conditions at GBs leaves residual CH_3_NH_2_ molecular entities incorporated into the Pb–I bonding network at the GB interface—a process-specific molecular defect class absent in solution-processed films [28].

Beyond chemical point defects, structural discontinuities at phase boundaries introduce lattice-strain-mediated defect states. In mixed-phase FAPbI_3_, the photoinactive *δ*-phase and photoactive *α*-phase coexist during thermal processing, creating *δ*/*α* interface regions with structurally frustrated Pb–I bonding configurations; this has been observed both in SVE-fabricated thin films on flexible substrates [29] and at single-crystal surfaces [30]. Halide compositional non-uniformity at GBs in FAPb(Br,I)_3_ leads to I-rich and Br-rich domain segregation under operating conditions, a dynamic and partially reversible process governed by illumination intensity and history, temperature, halide ratio, and dwell time [31,32]. Characterizing these diverse defect classes across multiple OHP compositions requires a probe with molecular vibrational specificity, which conventional electrical and optical methods cannot provide.

## 3. THz-TDS Methodology and the Lorentz Harmonic Oscillator Analysis

THz-TDS measurements were performed in transmission or reflection geometry using femtosecond-laser-based systems employing either photoconductive switching or optical rectification [33]. Depending on the experimental configuration, THz pulses were generated and detected with photoconductive antennas or electro–optic crystals driven by an 800 nm femtosecond laser (80 fs pulse duration, 80 MHz repetition rate). The spectral range covered 0.2–2.5 THz with a dynamic range > 40 dB, enclosed in a dry air-purged chamber to suppress atmospheric water vapor absorption. OHP thin films were deposited on Al_2_O_3_ substrates or flexible PET film and measured at room temperature without contacts or encapsulation.

The complex refractive index of the perovskite film $\tilde{n}\left(\omega \right)$ was extracted using an analytical formula of the complex transmission function $\tilde{T}\left(\omega \right)$ including multiple reflections [20]:

$$\tilde{T}\left(\omega \right)\;=\;\frac{{\tilde{E}}_{SAMPLE}}{{\tilde{E}}_{REF}}=\frac{2\tilde{n}({\tilde{n}}_{sub}+1)}{\left(1+\tilde{n})(\tilde{n}+{\tilde{n}}_{sub}\right)}\cdot exp\left[-i(\tilde{n}-1)\omega d/c\right]\cdot FP(\omega ),$$

with

$$P(\omega )=\frac{1}{1-\left(\frac{\tilde{n}-1}{\tilde{n}+1}\right)\left(\frac{\tilde{n}-{\tilde{n}}_{sub}}{\tilde{n}+{\tilde{n}}_{sub}}\right)\cdot exp\left[-2i\omega d\tilde{n}/c\right]}$$

where ${\tilde{E}}_{SAMPLE}$ and ${\tilde{E}}_{REF}$ denote the frequency-dependent THz electric fields transmitted through the sample and the reference substrate, respectively. ${\tilde{n}}_{sub}$ is the substrate refractive index, d is the film thickness, c is the speed of light in vacuum and $FP\left(\omega \right)$ is the Fabry–Perot effect term.

For thick samples, such as single crystals, for which transmission measurements are not feasible because THz radiation cannot penetrate the sample, the complex refractive index was determined from reflection-mode measurements. In this case, the reflected THz signal from the sample ${\tilde{E}}_{R,SAMPLE}\left(\omega \right)$ was compared with that from a gold-coated mirror ${\tilde{E}}_{R,REF}\left(\omega \right)$, which was treated as a perfect reflector in the THz frequency range. The complex reflectivity, $\tilde{r}\left(\omega \right)$, is expressed as [34]

$$\tilde{r}\left(\omega \right)=\frac{{\tilde{E}}_{R,SAMPLE}\left(\omega \right)}{{\tilde{E}}_{R,REF}\left(\omega \right)}=\frac{1-\tilde{n}}{1+\tilde{n}}=\left|R\right|e^{i\theta },$$

where *R* is the amplitude ratio and *θ* is the phase difference between the reflected THz fields from the sample and the reference mirror. The complex refractive index, $\tilde{n}$, can then be analytically obtained as

$$\tilde{n}=\left(\frac{1-R^{2}}{1+R^{2}-2Rcos\theta }\right)+i\left(\frac{2Rsin\theta }{1+R^{2}-2Rcos\theta }\right).$$

The complex optical conductivity $\tilde{\sigma }\left(\omega \right)$ was derived from the complex refractive index according to

$${\tilde{n}\left(\omega \right)}^{2}=\tilde{\epsilon }\left(\omega \right)=\epsilon_{\infty }+\frac{i\tilde{\sigma }\left(\omega \right)}{\omega \epsilon_{0}},$$

where $\epsilon_{\infty }$ is the high-frequency dielectric constant and $\epsilon_{0}$ is the vacuum permittivity.

The multi-oscillator Lorentz harmonic oscillator (LHO) model is extensively used to analyze the THz radiation response of perovskite materials because the major spectral features in this frequency range originate predominantly from carrier transport and phonon/defect resonances. In this model, each phonon contribution is treated as a damped harmonic oscillator, allowing quantitative determination of the resonance frequency, oscillator strength and scattering time. The multi-oscillator LHO model was fitted [20,30]:

$$\tilde{\sigma }\left(\omega \right)=-i\epsilon_{0}\omega \left(\epsilon_{\infty }-1\right)+\sum_{j}\frac{\epsilon_{0}\Omega_{j}^{2}\omega }{i\left(\omega_{0j}^{2}-\omega^{2}\right)+\omega \gamma_{j}},$$

where *ω*_0*j*_, Ω*_j_* and *γ_j_* are respectively the resonance frequency, oscillator strength, and scattering rate of the *j*-th mode. Defect modes are distinguished from intrinsic phonon modes by: (i) sensitivity to post-annealing treatment, (ii) correlation with XPS-quantified chemical defect concentration, and (iii) absence in solution-processed reference films.

### Quantities Directly Detected and Quantities Inferred

The quantity measured by THz-TDS is a vibrational resonance of the crystal lattice, and several conclusions drawn below rest on this distinction. THz-TDS directly detects (i) intrinsic phonon modes of the ABX_3_ framework, (ii) additional infrared-active vibrational modes that appear when the local bonding environment is perturbed, and (iii) free-carrier response. It does not detect a defect species as such. The chemical identity of a defect is inferred from the three criteria listed above, taken together with agreement between the measured and the calculated intrinsic phonon structure. A defect that produces no measurable lattice perturbation in this frequency window is THz-silent and will not appear in any spectrum reported here. Consequently, the absence of a defect-related THz feature constrains, but does not establish, the absence of defects.

It follows that the oscillator strength Ω is not a direct count of defect sites. Ω measures the infrared activity generated by defect-induced distortion of the Pb–X framework and therefore reflects the product of defect concentration and the strength of the mechanical coupling between the defect and the host lattice. Where a linear relation between Ω and an XPS-determined concentration has been established for a given material and process, Ω functions as a calibrated proxy for that concentration. Such a calibration is material- and process-specific and cannot be transferred between compositions without re-derivation.

## 4. THz Defect Fingerprint Library Across OHP Compositions

### 4.1. MAPbI_3_: The 1.58 THz Fingerprint of CH_3_NH_2_ Grain-Boundary Defects

**Figure 1:**
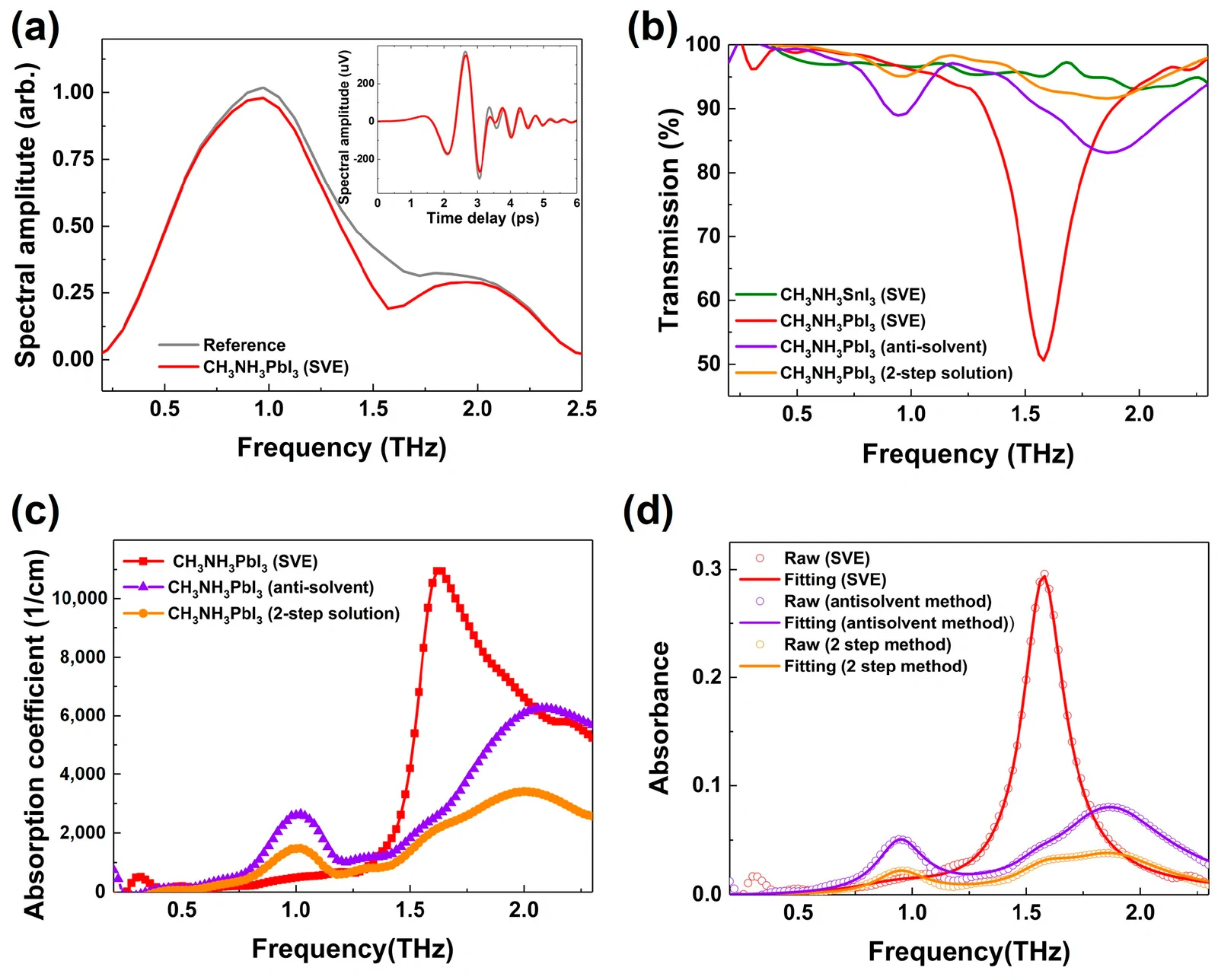
THz characterization of MAPbI_3_ thin films prepared by different fabrication methods. (**a**) THz waveforms and (**b**) transmission spectra: the SVE film uniquely shows ~50% suppression at 1.58 THz. (**c**) Absorption coefficient spectra (>11,000 cm^−1^ at 1.58 THz for SVE). (**d**) THz absorbance decomposed into three Lorentz oscillators at 0.95, 1.58, and 1.87 THz. Reproduced from Maeng et al., *Sci. Rep.* **2019**, *9*, 5811 [28].

The most definitive example of a process-specific THz defect fingerprint was established in 2019 by Maeng et al. [28], who showed that SVE-fabricated MAPbI_3_ thin films exhibit a strong, narrow absorption at 1.58 THz with an absorption coefficient exceeding 11,000 cm^−1^—approximately two orders of magnitude above the background phonon level. Figure 1 compares the THz transmission spectra of MAPbI_3_ fabricated by SVE, a two-step solution, an anti-solvent, and MASnI_3_. Only the SVE sample shows the 1.58 THz absorption, accounting for ~50% suppression of THz transmission at that frequency. LHO fitting resolves three oscillators at 0.95, 1.58, and 1.87 THz, where the 0.95 and 1.87 THz modes are present in all samples as intrinsic lattice phonons, while the 1.58 THz oscillator is exclusive to SVE fabrication.

**Figure 2:**
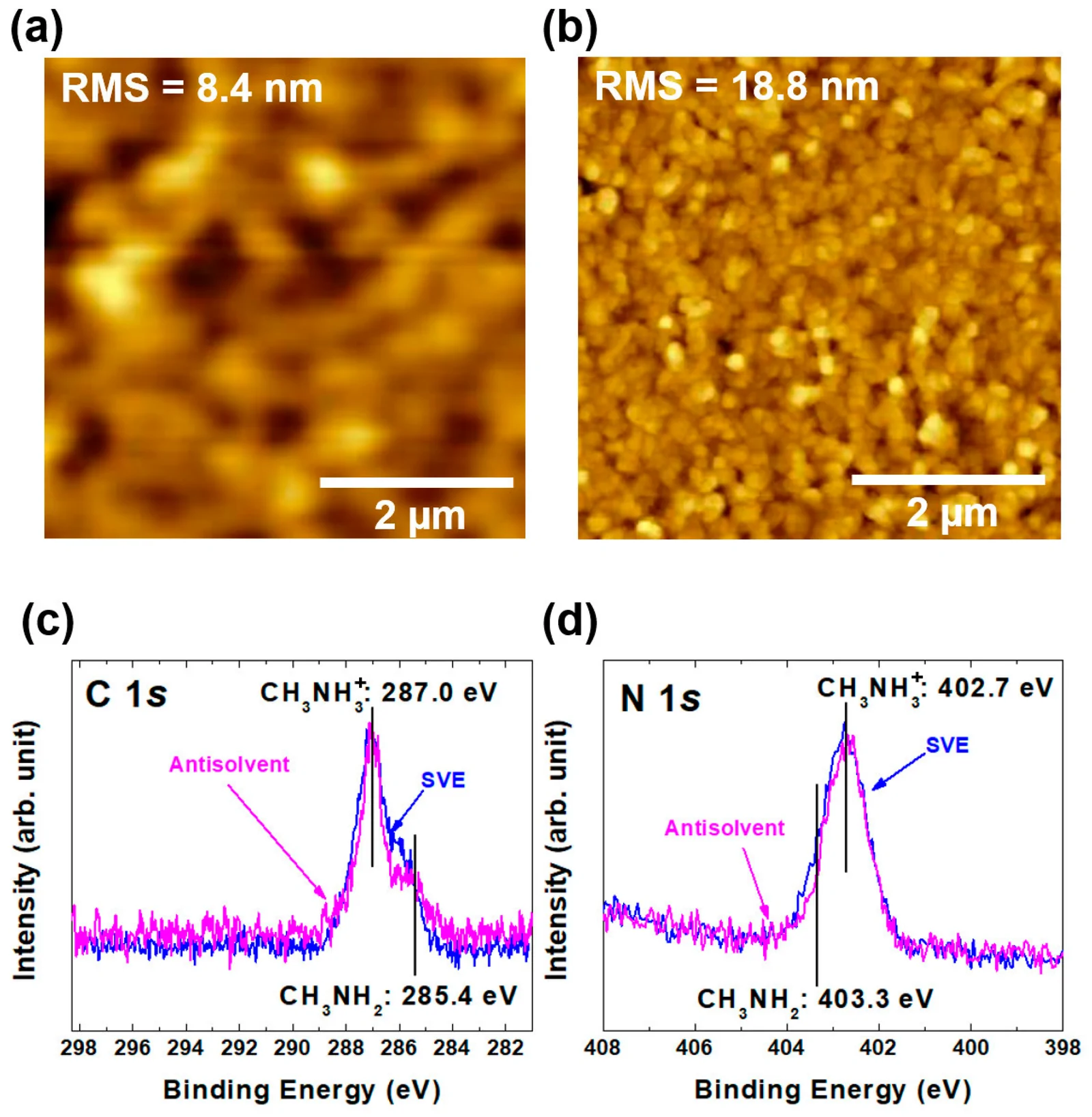
XPS and surface morphology evidence for CH_3_NH_2_ grain-boundary defects. (**a**,**b**) SEM images: SVE (RMS = 18.8 nm) vs. anti-solvent film (RMS = 8.4 nm). (**c**,**d**) C and N 1*s* XPS spectra confirming elevated CH_3_NH_2_^+^ intensities at 287.0 eV and 402.7 eV in SVE film(blue) and anti-solvent film(magenta). Reproduced from Maeng et al., *Sci. Rep.* **2019**, *9*, 5811 [28].

The density function theory (DFT) phonon calculations on tetragonal CH_3_NH_3_PbI_3_ reproduce the 0.95 and 1.87 THz intrinsic modes but not the 1.58 THz peak, directly pointing to a defect-related origin. XPS characterization confirms higher CH_3_NH_2_ molecular density at grain boundaries in SVE films: C and N 1*s* core-level spectra show elevated intensities at 287.0 eV and 402.7 eV (CH_3_NH_2_^+^ binding energies), absent in anti-solvent films. SEM reveals smaller grains and higher GB density in SVE films (RMS roughness 18.8 nm vs. 8.4 nm) (Figure 2). The 1.58 THz mode is thus assigned to vibration of the Pb–I bond structurally deformed by the co-incorporated CH_3_NH_2_ molecular defect at GB sites [20,21].

### 4.2. Quantitative Correlation: THz Oscillator Strength as a Calibrated Defect-Density Proxy

Maeng et al. (2020) [35] established the quantitative value of the 1.58 THz fingerprint by systematically varying CH_3_NH_2_ defect density in MAPbI_3_ through post-annealing under a N_2_ atmosphere and correlating results with XPS-derived defect concentrations. Four MAPbI_3_ samples (A–D) were prepared at different annealing conditions: no annealing (A), 110 °C/45 min (B, optimal), 150 °C/10 min (C), and 150 °C/30 min (D, over-annealed).

**Figure 3:**
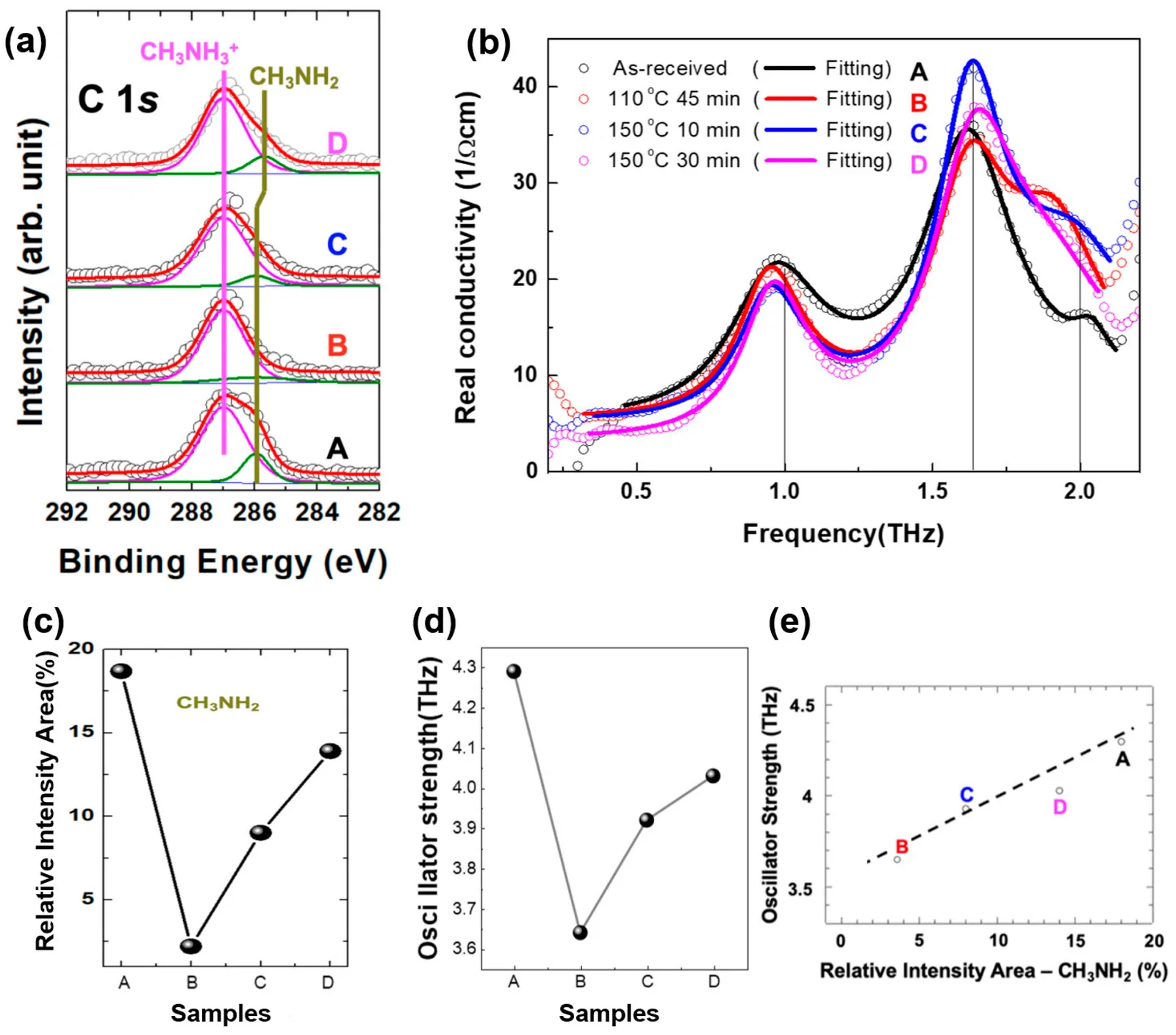
Quantitative defect fingerprinting in MAPbI_3_. (**a**) C 1*s* spectra and curve-fitting results for samples A–D. Experimental data are shown as dots, and the fitted curves are shown as red lines. The component assignments include CH_3_NH_3_^+^ (magenta lines) and CH_3_NH_2_ molecular defects (green lines). (**b**) THz conductivities of samples A–D. (**c**) Relative CH_3_NH_2_ peak intensities (%) for the chemical states of the samples. (**d**) THz oscillator strength at 1.6 THz. (**e**) Linear correlation between (**c**,**d**). Reproduced from Maeng et al., *Nanomaterials* **2020**, *10*, 721 [35].

Figure 3 shows the curve-fitting results of C 1*s* core-level spectra and extracted CH_3_NH_2_ relative intensities across samples A–D. The CH_3_NH_2_ defect concentration follows a non-monotonic trend: high in sample A (~18%), nearly eliminated in sample B (~1%), and then recovered in C and D (~15%) as over-annealing decomposes the perovskite. LHO fitting yields oscillator strengths at 0.97, 1.62, and 2.01 THz. Only the 1.62 THz oscillator strength reproduces the same non-monotonic pattern—in direct, linear proportion to XPS-quantified CH_3_NH_2_ defect concentration. The 0.97 and 2.01 THz modes remain constant, confirming their intrinsic phonon origin.

This linear THz oscillator strength–defect concentration relationship establishes THz-TDS as a quantitative, contact-free proxy for defect concentration that bypasses the surface-depth limitation of XPS and the species-averaging limitation of PL [35]. Eliminating this defect (Sample B, Ω (1.6 THz) ≈ 0) directly correlates with recovery of full OHP crystal structure and optimal device performance. This constitutes Pillar I of the three-pillar framework; the statistical limitations of the present calibration are assessed in Section 5.1.

### 4.3. MAPbBr_3_: Phonon Fingerprints Insensitive to Grain-Boundary Molecular Chemistry

**Figure 4:**
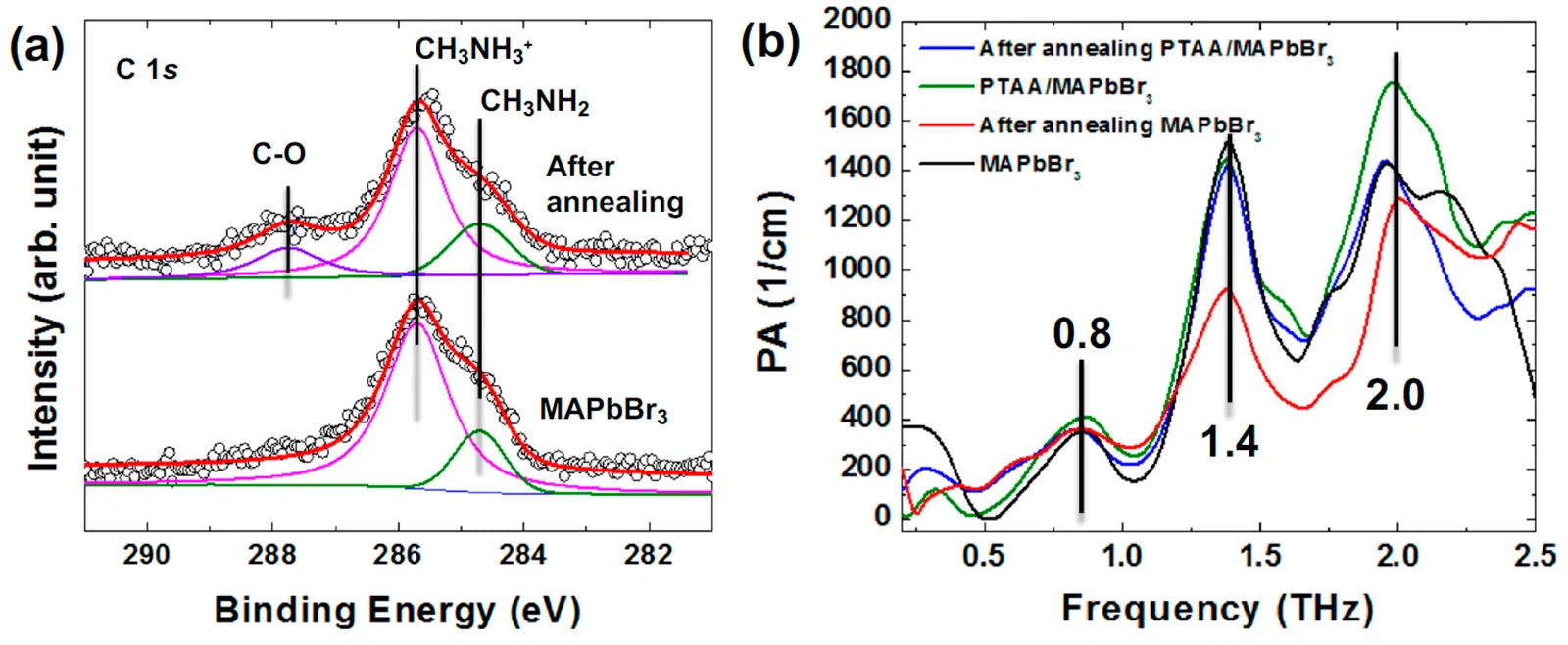
THz phonon fingerprints of SVE-fabricated MAPbBr_3_. (**a**) C 1*s* core-level spectra and curve-fitting results before and after 150 °C annealing. Experimental data are shown as dots, and the fitted curves are shown as red lines. The component assignments include C–O surface impurities (purple lines), CH_3_NH_3_^+^ (magenta lines) and CH_3_NH_2_ molecular defects (green lines). The CH_3_NH_2_ molecular defect component (287.0 eV) is substantially reduced after annealing. (**b**) THz absorption spectra: three phonon modes at 0.8, 1.4, and 2.0 THz unchanged by defect chemistry modification. Reproduced from Maeng et al., *NPG Asia Mater.* **2020**, *12*, 53 [36].

Maeng et al. (2020) [36] demonstrated that SVE-fabricated MAPbBr_3_ shows three sharp, stable phonon absorptions at 0.8, 1.4, and 2.0 THz that are completely insensitive to grain-boundary CH_3_NH_2_ defect chemistry. Despite XPS confirming substantial CH_3_NH_2_ reduction upon post-annealing, the THz phonon modes remain unchanged in frequency, oscillator strength, and linewidth (Figure 4). This contrast with MAPbI_3_ is mechanistically explained by the stronger Pb–Br bond, which resists structural deformation upon CH_3_NH_2_ incorporation and thus does not produce a defect-induced vibration shift.

**Figure 5:**
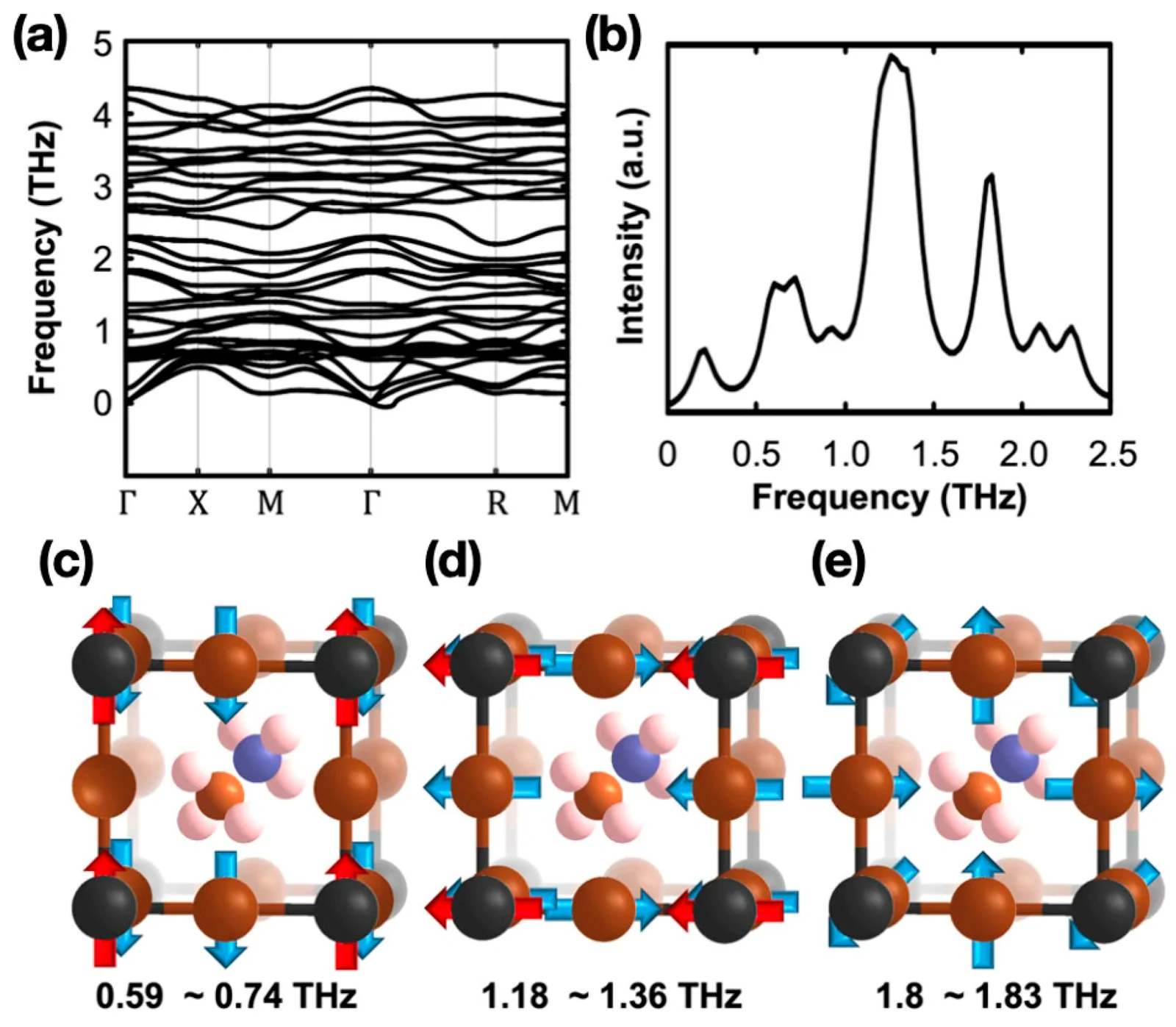
TDEP-DFT phonon analysis of MAPbBr_3_. (**a**) Phonon dispersion at 300 K with three IR-active branches. (**b**) Simulated IR absorption vs. experimental THz conductivity. (**c**–**e**) Phonon eigenvectors at 0.8, 1.4, and 2.0 THz. The corresponding real-space vibrational modes are associated with Pb–Br–Pb transverse vibrations, Pb–Br–Pb longitudinal optical vibrations, and optical Br vibrations, respectively. Grey, brown, purple, orange, and pink spheres denote Pb, Br, C, N, and H atoms, and the arrows indicate the phonon eigenvectors. Reproduced from Maeng et al., *NPG Asia Mater.* **2020**, *12*, 53 [36].

First-principles phonon calculations using the temperature-dependent effective potential (TDEP) method on cubic MAPbBr_3_ at 300 K (Figure 5) [36] assign the three modes as: 0.8 THz—transverse vibration of the MA cation; 1.4 THz—longitudinal optical vibration of the Pb–Br framework; 2.0 THz—self-vibration of the Br sublattice. This DFT-level assignment provides a rigorous quantum-mechanical basis for the THz fingerprint assignments.

MAPbBr_3_ thus defines the ‘clean phonon’ regime of the OHP THz fingerprint library, within bounded limits: under the annealing conditions tested and within the sensitivity of the reported measurement, no defect-induced THz mode was detected, and the THz spectrum matches the DFT-predicted intrinsic phonon structure. This is a statement about the absence of a detectable THz-active molecular defect mode, not about the absence of defects. THz-silent defect states, including charged point defects that produce negligible lattice distortion, may be present and would require complementary techniques to detect.

### 4.4. FAPbI_3_: Phase-Boundary Phonon Fingerprints

Phase control is a central challenge for FA-based perovskites: the photoactive *α*-FAPbI_3_ (black cubic phase) is thermodynamically metastable at room temperature and reverts to the photoinactive *δ*-phase (yellow hexagonal phase) unless stabilized. THz-TDS provides a direct, non-destructive window into phase-boundary formation that complements XRD, offering sensitivity to the interfacial bonding state rather than the bulk crystal structure. Two sample geometries have been examined.

Lee et al. (2019) [29] showed that SVE-fabricated FAPbI_3_ thin films on flexible ITO-coated PEN, annealed at 140 °C for 10 min, form a mixed state with a major *δ*-phase and a minor *α*-phase, confirmed by XRD and by the optical bandgap shift from 1.81 to 1.57 eV. This mixed state exhibits a single THz absorption at 1.62 THz with approximately 40% absorptance. The feature is absent in the as-deposited *δ*-phase and disappears after 20 min or more of annealing, when XRD indicates the pure *α*-phase. C 1*s* core-level spectra resolve two chemical states corresponding to *δ*-FA^+^ (287.9 and 284.7 eV) and *α*-FA^+^ (286.3 and 284.3 eV) with an intensity ratio near 5:1, whereas N 1*s*, Pb 4*f*, and I 4*d* spectra are unchanged. The 1.62 THz mode was assigned to a Pb–I vibration of the *α*-phase regions embedded in the main *δ*-phase structure.

**Figure 6:**
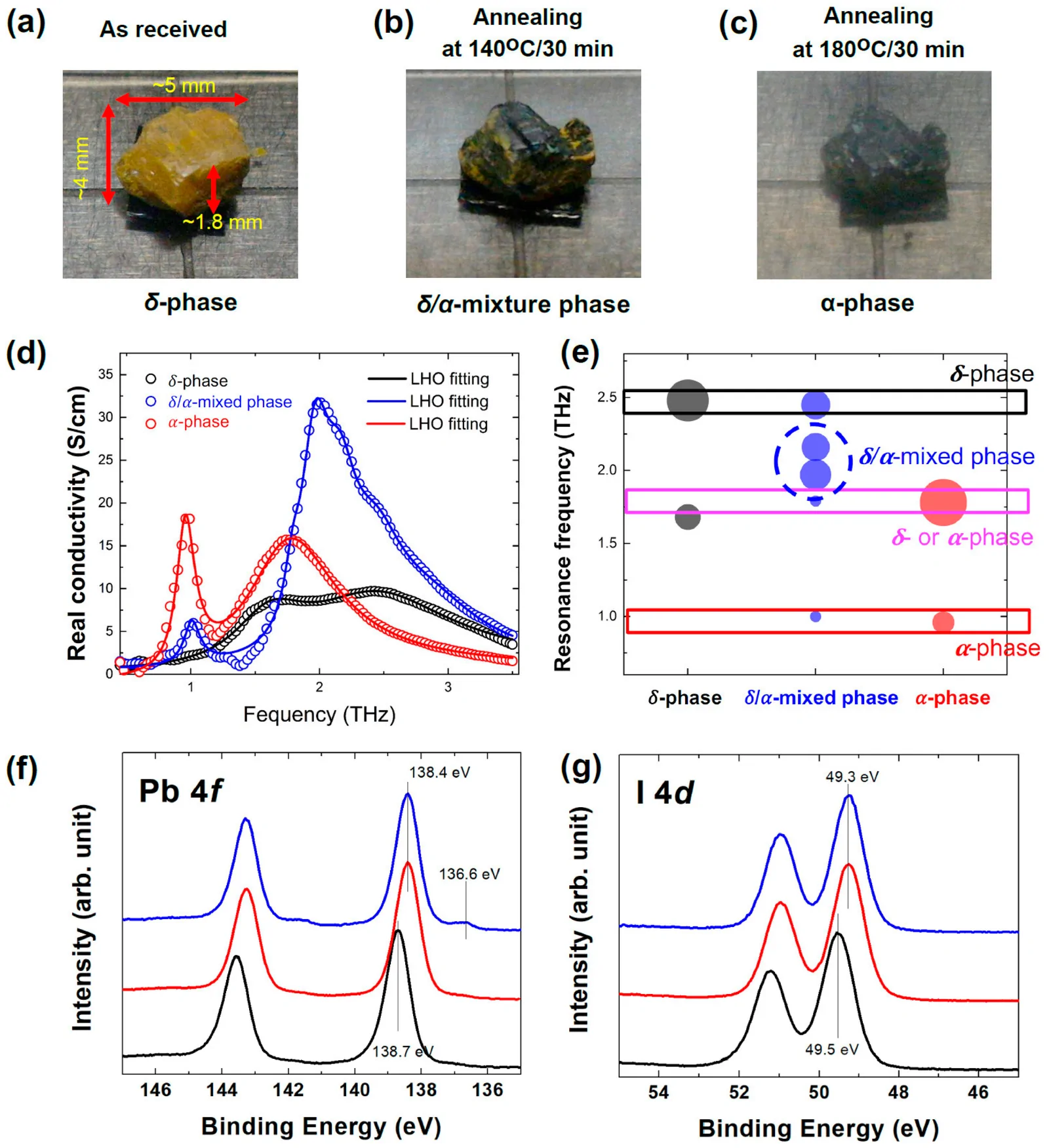
Phase-sensitive THz fingerprints in FAPbI_3_. (**a**–**c**) Surface morphology: *δ*-phase (as-deposited), *δ*/*α*-mixed phase (140 °C/30 min), *α*-phase (180 °C/30 min). (**d**) THz real conductivities of *δ*-, δ/α-mixed and α-phase FAPbI_3_ single crystals and its Lorentz harmonic oscillator (LHO) fittings. (**e**) The fitting parameters of LHO model. The mixed phase uniquely shows 2.0 and 2.2 THz modes. (**f**,**g**) Pb 4*f* and I 4*d* core-level spectra. The δ-, δ/α-mixed-, and α-phase spectra are shown as black, blue, and red lines, respectively. Pb 4*f* and I 4*d* XPS confirming phase identity. Reproduced from Maeng et al., *NPG Asia Mater.* **2021**, *13*, 75 [30].

Maeng et al. (2021) [30] demonstrated that δ/α-mixed-phase FAPbI_3_ single-crystal surface regions, formed by controlled annealing at 140 °C for 30 min, exhibit two unique THz absorption peaks at 2.0 and 2.2 THz that are absent from both pure *δ*- and *α*-phases (Figure 6). The pure *α*-phase (180 °C) shows a single phonon mode at ~1.45 THz. Phase assignments were confirmed by Pb 4*f* and I 4*d* core-level spectra, which show no new chemical states at the mixed-phase boundary.

Molecular dynamics (MD) simulations—employed here instead of static DFT to capture the thermal disorder inherent to the *δ*/*α*-mixed-phase interface at finite temperature—confirm that the 2.0 and 2.2 THz modes originate from compressed Pb–I bond lengths at the structurally frustrated *δ*/*α* interface layer. These interfacial phonon modes are: (i) upshifted from the bulk *α*-phase 1.45 THz mode by bond compression, (ii) invisible to XRD, and (iii) uniquely correlated with the presence of a seamless phase boundary. They constitute a THz fingerprint of phase-boundary structural integrity.

The two geometries agree that a THz-active vibration exists only when the two phases coexist and that it is invisible to XRD, but they disagree on frequency, and this discrepancy remains unresolved. Plausible contributions include the different *δ*:*α* ratios of the two samples, the different dimensionality and strain state of a film on a flexible polymer substrate compared with a crystal surface, and the different annealing durations. Until this is settled, the interfacial mode should be treated as a qualitative indicator of phase-boundary formation rather than as a transferable quantitative fingerprint.

**Figure 7:**
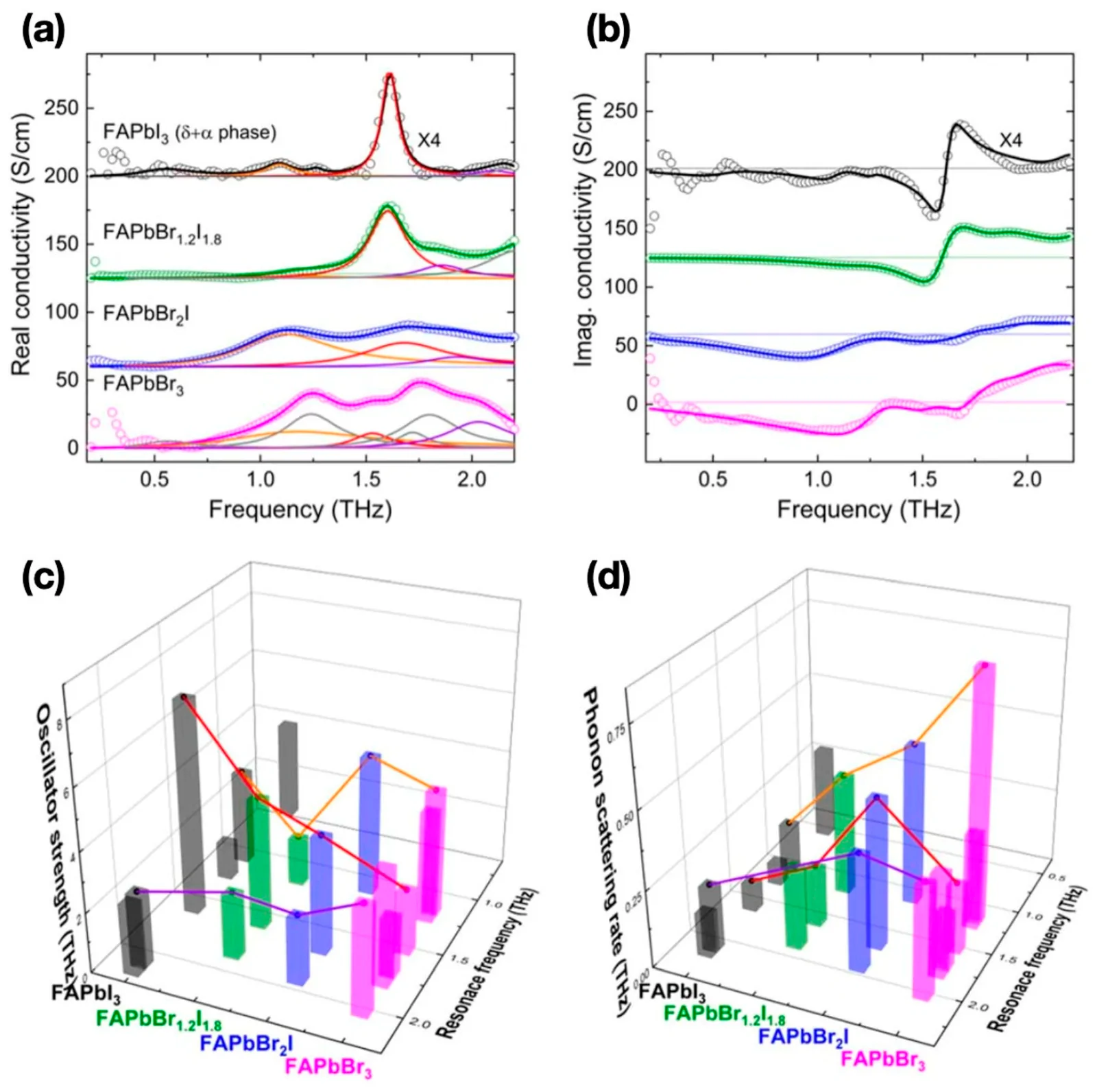
Composition-dependent THz fingerprints in FAPb(Br_x_I_1−x_)_3_ thin films fabricated by SVE. THz conductivity spectra across the composition series: I-rich compositions show large ~1.6 THz GB absorption; Br-rich compositions converge on three intrinsic phonon modes. (**a**,**b**) Real and imaginary conductivities of FAPb(Br_x_I_1−x_)_3_ films (dots) and its Lorentz harmonic oscillator fitting (lines). (**c**,**d**) Correlations of fitting parameters with resonance frequency for different I/Br concentration ratios. Reproduced from Maeng et al., *Appl. Phys. Express* **2021**, *14*, 121002 [32].

In mixed-halide FAPb(Br_x_I_1−x_)_3_, the THz fingerprint is tunable by composition [32]. I-rich formulations (x ≤ 0.3) show strong absorption at ~1.6 THz from I-rich GB domains, while Br-rich compositions (x ≥ 0.5) transition toward three-phonon behavior (Figure 7). This composition–THz fingerprint map directly guides halide optimization for minimizing I-related GB defect density in FA-based tandem cells [31].

Two qualifications apply. First, these measurements were made on as-prepared films in the dark at room temperature and therefore characterize the as-grown compositional state; they should not be read as describing the segregated state that develops under operation, whose extent and timescale depend on illumination intensity and history, temperature, halide ratio, grain size, and dwell time under measurement [31]. Second, resolving that segregated state would require THz measurement under illumination with controlled soaking time and temperature, which to our knowledge has not yet been reported for this system.

### 4.5. γ-CsPbI_3_: Grain-Size-Independent Phonon Fingerprints

The all-inorganic *γ*-CsPbI_3_ (orthorhombic, black phase) is thermally stable compared to organic-cation analogs. The absence of CH_3_NH_2_ decomposition products at grain boundaries provides a direct test of whether the 1.58 THz defect fingerprint in SVE-fabricated films is uniquely tied to organic MA cation chemistry.

**Figure 8:**
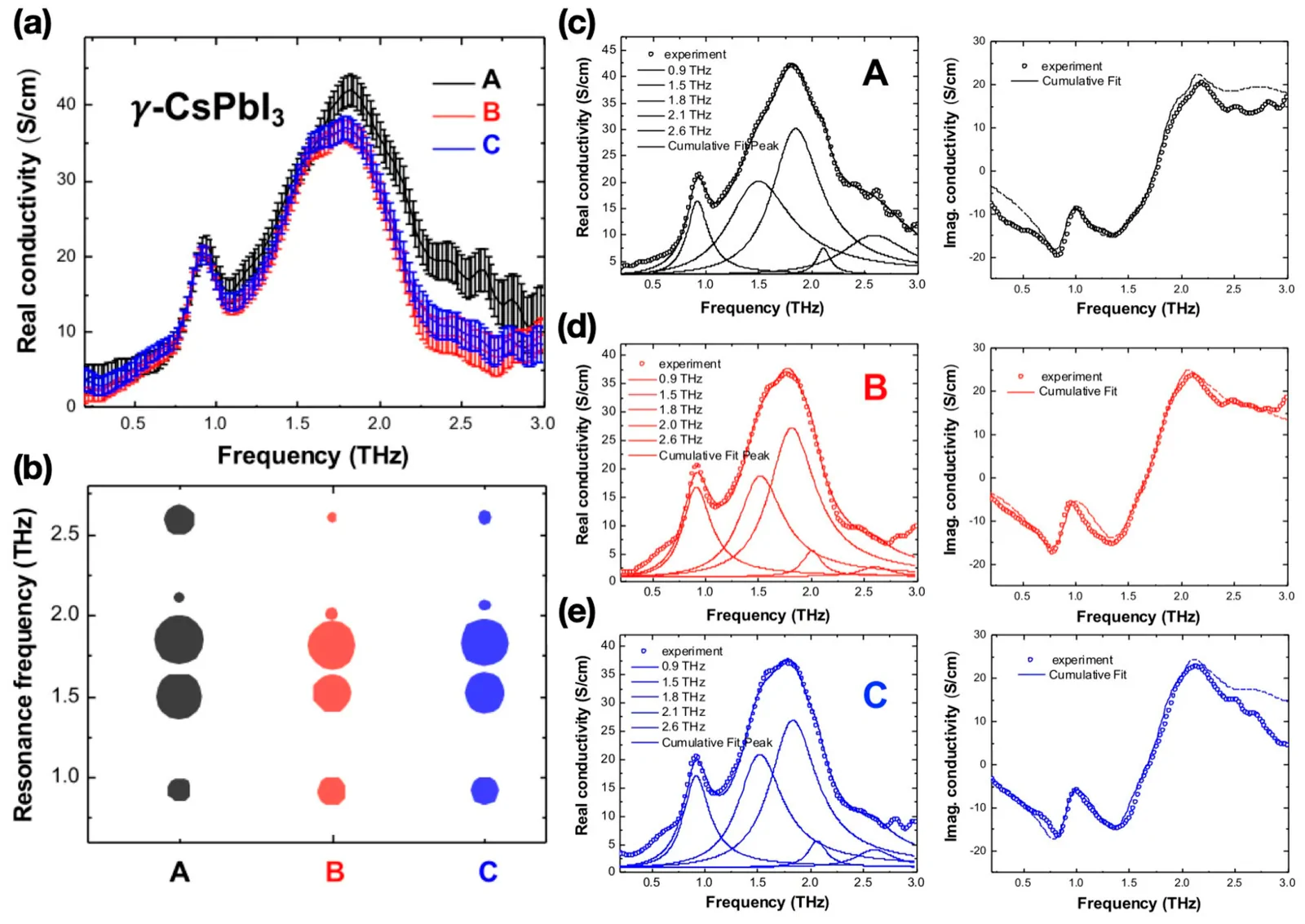
Grain-size-independent THz phonon fingerprints in *γ*-CsPbI3 thin films. (**a**) Real conductivity for samples A (300 nm), B (350 nm), and C (460 nm): identical three-phonon spectra. (**b**) Resonance frequencies and oscillator strengths of Lorentz harmonic oscillator fitting as a function of grain size. The size of each dot is proportional to the oscillator strength. No grain-size dependence is observed. (**c**–**e**) Individual LHO component fits for A, B, and C. Reproduced from Maeng et al., *Mater. Today Phys.* **2023**, *30*, 100960 [37].

Maeng et al. (2023) [37] examined solution-processed *γ*-CsPbI_3_ films with three grain sizes (A: 300 nm, B: 350 nm, C: 460 nm) and measured THz conductivity (Figure 8). All three samples show three clean phonon absorption peaks at 0.9, 1.5, and 1.8 THz with no additional absorption. Both resonance frequencies and oscillator strengths are entirely grain-size-independent. DFT phonon calculations assign the three modes as: 0.9 THz (transverse I–Pb–I framework vibration), 1.5 THz (Cs–I–Cs optical vibration), and 1.8 THz (longitudinal I–Pb–I framework vibration).

Two constraints bound this interpretation. First, these films were solution-processed, whereas the MAPbI_3_ comparison films that carry the 1.58 THz mode were fabricated by SVE. The comparison therefore varies the composition and fabrication method simultaneously, and the absence of a defect mode in *γ*-CsPbI_3_ cannot be attributed to composition alone; a directly matched SVE-fabricated *γ*-CsPbI_3_ series would be required to isolate the process contribution. Second, the grain-size interval tested spans only 300–460 nm. The supported conclusion is that no grain-size-dependent THz defect resonance was detected over this interval, and that the measured spectrum is consistent with the DFT-predicted intrinsic phonon structure; chemically active but THz-silent defects may nonetheless be present. Subject to these constraints, the contrast between the defect-active 1.58 THz mode in SVE-fabricated MAPbI_3_ and the clean three-phonon spectrum of *γ*-CsPbI_3_ remains consistent with, and supportive of, a molecular-chemistry-specific origin for the 1.58 THz signal rather than a structural GB artifact.

## 5. The Three-Pillar THz Defect Engineering Framework

**Figure 9:**
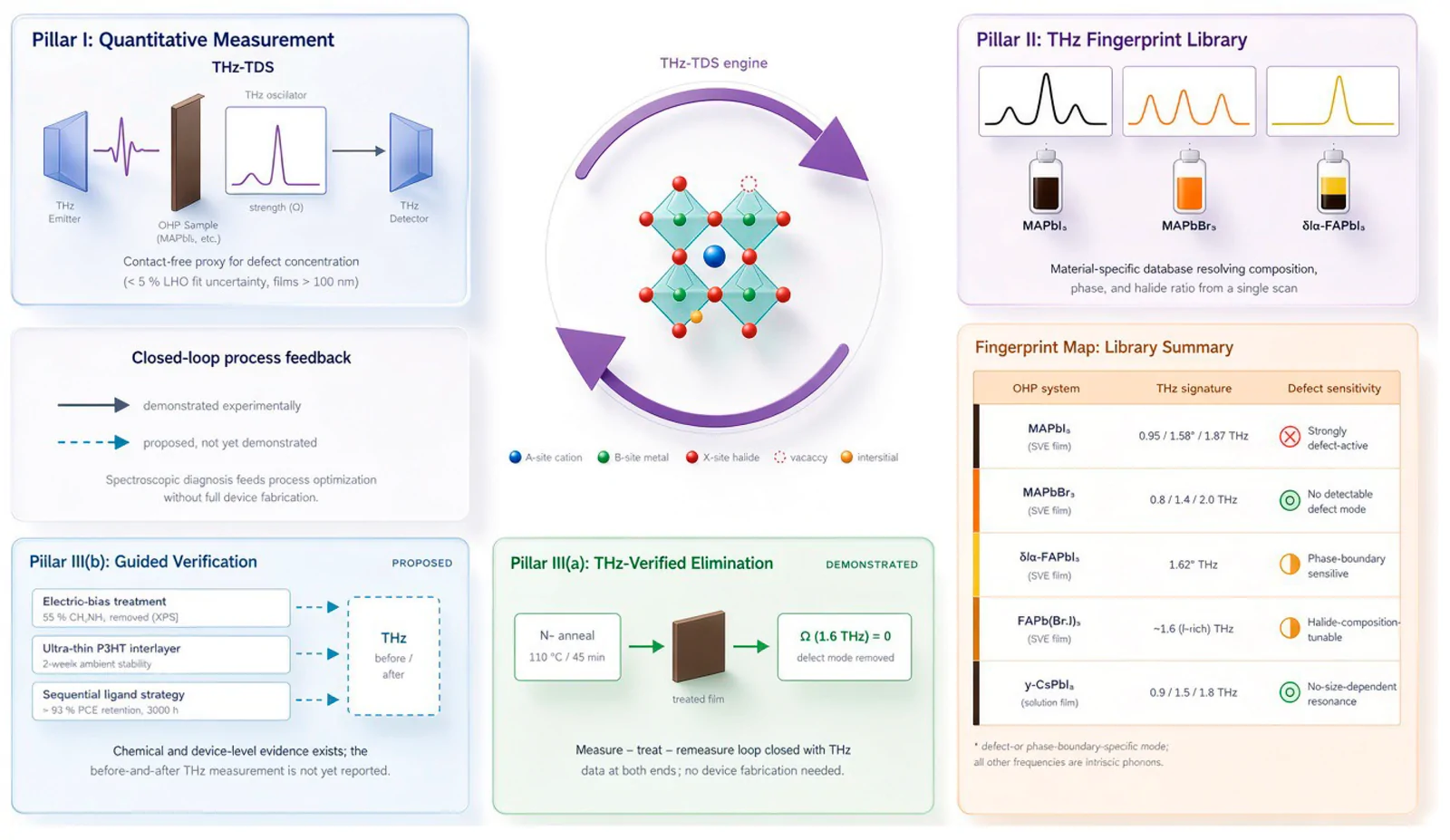
Schematic of the three-pillar THz defect engineering framework. Three interconnected pillars—(I) quantitative defect measurement, (II) material-specific THz fingerprint library, and (III) fingerprint-guided defect elimination—operate in a continuous cycle around the central THz-TDS engine, converting raw spectral data into actionable process feedback for OHP solar cell manufacturing. Pillar III is shown in two parts: III (a), the measure–treat–remeasure loop already closed with THz data at both ends, and III (b), treatments for which chemical or device-level evidence exists but the before-and-after THz measurement has not yet been reported. Solid arrows denote steps demonstrated experimentally; dashed arrows denote proposed extensions not yet demonstrated. The library summary reproduces the sample form, processing route, and mode assignments of Table 1.

The THz fingerprint library presented above directly enables a three-pillar engineering framework that links spectroscopic defect identification to process optimization and device performance enhancement. Figure 9 illustrates the interconnected cycle: Pillar I provides quantitative measurement capability, Pillar II supplies compositional context, and Pillar III closes the engineering loop through targeted defect elimination. Pillars I and II rest on published measurements; Pillar III is partly demonstrated and partly proposed, and the distinction is made explicit in Section 5.3.

### 5.1. Pillar I—Quantitative Defect Measurement

The linear correlation between oscillator strength Ω (1.6 THz) and XPS-quantified CH_3_NH_2_ concentration [35] transforms THz oscillator strength into a non-contact defect concentration meter. The measurement uncertainty, estimated from repeated LHO fits on identically prepared films, is <5% for films >100 nm [35]; spectral isolation of the 1.6 THz mode from the 0.97 and 2.0 THz phonons enables unambiguous LHO decomposition; and ambient room-temperature measurement eliminates the vacuum-sample-transfer requirement of XPS. This enables: (i) substrate-by-substrate, process-step-resolved defect quantification; (ii) inline monitoring of post-annealing conditions without device fabrication; and (iii) tracking of defect-density evolution across roll-to-roll SVE production sequences.

The scope of this calibration is nevertheless narrow: it rests on four annealing conditions (samples A–D) within a single material system. Regression statistics, replicate counts, per-point error bars, and a limit of detection have not been reported, and the quantity calibrated is the relative XPS C 1*s* CH_3_NH_2_ intensity rather than an absolute defect areal density, so the relation is a proportionality rather than an absolute scale. Establishing THz-TDS as a quantitative defect metrology tool in the full sense will require, at minimum: a calibration series with at least eight to ten independently prepared conditions; replicate films at each condition with reported standard deviations; a regression coefficient with confidence intervals; a stated limit of detection expressed in oscillator-strength units and converted to concentration; and a thickness-dependence study establishing the valid film-thickness window.

### 5.2. Pillar II—Universal THz Fingerprint Library

The five OHP systems reviewed define a material-specific THz fingerprint library (Table 1) spanning defect-active, defect-quiet, phase-sensitive, and composition-tunable regimes. This library enables spectroscopic discrimination of: (i) fabrication method (SVE vs. solution-processed, through presence/absence of the 1.58 THz mode); (ii) OHP composition (distinct phonon frequencies for MA, FA, Cs); (iii) crystallographic phase (*δ* vs. *α* vs. *δ*/*α*-mixed through 1.45 vs. 2.0/2.2 THz modes); and (iv) halide composition (THz oscillator-strength map for FAPb(Br,I)_3_ series). No single conventional technique provides this combination of information non-destructively from a single measurement.

Three boundaries currently delimit this library: it spans five compositions, draws predominantly on a single fabrication route, and originates largely from one laboratory. General applicability across the halide perovskite family therefore remains a target rather than an established property, and reaching it requires extension to further compositions, processing routes, and independent laboratories.

**Table 1:** THz defect fingerprint library for the OHP systems reviewed. Sample form and processing route are stated for each entry, since both influence the observed spectrum.

OHP System	Sample Form/Processing	THz Modes (THz)	Defect Sensitivity	Physical Assignment	Independent (Non-Author-Group) Validation	Ref.
MAPbI_3_	Thin film; SVE (vs. two-step solution and anti-solvent references)	0.95/1.58 */1.87	Strongly defect-active (1.58 THz)	* CH_3_NH_2_ mol. defect at GB; others = bulk phonons	Intrinsic phonon frequencies consistent with [20,21]; 1.58 THz defect mode not independently reproduced	[28,35]
MAPbBr_3_	Thin film; SVE	0.8/1.4/2.0	No detectable defect-induced mode under conditions tested	All intrinsic phonons (TDEP-DFT-verified)	Optical phonon frequencies consistent with [21,22]; negative result not independently reproduced	[36]
*δ*/*α*-FAPbI_3_	Thin film; SVE on flexible ITO/PEN	1.62 *	Phase-boundary-sensitive	* Pb–I vibration of *α*-phase regions in *δ* matrix	Not independently reproduced	[29]
*δ*/*α*-FAPbI_3_	Single crystal, surface region	1.45/2.0 */2.2 *	Phase-boundary-sensitive	* Interfacial phonons at seamless *δ*/*α* boundary	Not independently reproduced	[30]
FAPb(Br_x_I_1−x_)_3_	Thin film; SVE	Composition-dep.	Halide-composition-tunable	I-rich: GB absorption; Br-rich: intrinsic phonons	Segregation behavior independently established [31]; THz map not reproduced	[32]
*γ*-CsPbI_3_	Thin film; solution-processed; grains 300/350/460 nm	0.9/1.5/1.8	No grain-size-dependent resonance over 300–460 nm	0.9 transverse I–Pb–I; 1.5 Cs–I–Cs optical; 1.8 longitudinal I–Pb–I	Not independently reproduced	[37]

Asterisk (*) denotes defect- or phase-boundary-specific modes.

### 5.3. Pillar III—Fingerprint-Guided Defect Elimination

The THz fingerprint provides immediate, quantitative feedback for elimination strategies where before-and-after THz data exist, as summarized in Table 2. Thermal post-annealing at 110 °C for 45 min in N_2_ reduces THz oscillator strength Ω (1.6 THz) from ~0.37 to near zero in MAPbI_3_ [35]; this is the one case in which the full measure–treat–remeasure loop has been closed with THz data at both ends. For the remaining strategies the evidence is chemical or device-level rather than spectroscopic. Electric-bias treatment (15 V) of MAPbBr_3_ single-crystal surfaces eliminates 55% of CH_3_NH_2_ through field-driven ionic migration, as established by XPS [38]. Ultra-thin P3HT (7 nm) HTL deposition creates a chemically clean interface and provides 2-week air stability protection [39]. Advanced sequential ligand strategies such as the two-step dedicated-ligand approach by Ahmad et al. [14]—applying DFABr for grain boundaries and 4APPBr_2_ for grain surfaces—have demonstrated >93% PCE retention after 3000 h under high humidity. Applying THz defect fingerprinting before and after such treatments would directly quantify elimination efficacy, providing a spectroscopic certificate of passivation. Such before-and-after measurements have not yet been reported, and they constitute the most immediate experimental extension of this framework.

## 6. Discussion

The THz defect fingerprint approach occupies a unique position in the OHP characterization landscape. Early OPTP-THz studies focused on carrier mobility and dynamics [19], establishing THz-TDS as a benchmark for transport quality but not defect identity. Temperature-dependent Time-Resolved Terahertz Spectroscopy (TRTS) by Milot et al. [40] demonstrated how charge-carrier recombination and mobility in MAPbI_3_ evolve across phase transitions. Transmission THz-TDS studies by La-o-vorakiat et al. [20] and Sendner et al. [21] mapped intrinsic phonon modes and their coupling to carrier transport, and these independent datasets provide the external corroboration for the intrinsic phonon assignments used throughout Section 4. Near-field THz nanoimaging by Kim et al. [23] spatially resolved GB trap filling with <20 nm resolution—a nanoscale complement to the far-field oscillator-strength approach. Hempel et al. [41] demonstrated that THz and microwave transient measurements alone can quantitatively predict PCE of perovskite solar cells, bridging the gap between non-contact spectroscopy and device-level benchmarking.

Table 3 compares THz-TDS against principal competing characterization techniques; the entries describe standard laboratory implementations, and advanced implementations of several techniques extend beyond what is tabulated. Compared to PL: THz oscillator strength is immune to reabsorption, surface passivation artifacts, and excitation-dependent injection. Compared to impedance spectroscopy: THz-TDS requires no contacts and accesses THz-frequency lattice dynamics inaccessible to kHz–MHz impedance measurements. Compared to XPS: THz-TDS is non-destructive and non-contact, operates under ambient conditions, and probes the entire film depth, but it identifies chemical species only indirectly and requires XPS for its own calibration.

The distinct response of the 1.58 THz mode in MAPbI_3_ compared to the unchanged phonon spectrum of MAPbBr_3_ provides a critical physical insight into the lattice-defect interaction [28,36]. While both systems were fabricated via SVE, the contrast originates from the disparity in Pb–X (X = I, Br) bond energies. The stronger ionic bonding and higher lattice rigidity of the bromide framework suppress structural deformations induced by incorporated CH_3_NH_2_ molecular defects [36]. In contrast, the relatively soft Pb–I lattice in MAPbI_3_ is more susceptible to local distortions, which activate the specific vibrational mode observed at 1.58 THz [28]. THz-TDS therefore probes not merely the presence of defects but the mechanical coupling between the defect species and the host lattice, providing a spectroscopic gauge of lattice stability.

Phase segregation under illumination in mixed-halide OHPs—documented by Motti et al. [31] and others—is directly observable in the THz composition-fingerprint map: I-rich GB domains correspond to elevated Ω (1.6 THz), while Br-rich compositions maintain phonon-dominated spectra. Xu et al. [42] recently demonstrated real-time detection of MAPbI_3_ aging via monotonic decay of a THz absorption peak at 0.968 THz, tracking degradation onset non-destructively—consistent with the fingerprint-guided monitoring concept presented here.

Correlating surface-sensitive XPS with bulk-probing THz oscillator strength presupposes a depth-uniform defect distribution. SVE plausibly satisfies this condition: whereas solvent evaporation in solution processing can generate vertical composition gradients, sequential vacuum evaporation distributes trapped molecular species more homogeneously through the film thickness [35]. On this basis, the surface CH_3_NH_2_ concentration approximates the GB defect density of the full film, supporting the use of the 1.6 THz oscillator strength as a contact-free bulk proxy for quality control [35]. Depth-resolved profiling would be required to test this assumption directly.

### 6.1. Dynamic Defects and Measurement Under Operating Conditions

Defects in halide perovskites are dynamic. Populations and spatial distributions change under illumination, applied bias, elevated temperature, and prolonged operation, and *in situ/operando* methods have become essential for capturing this behavior [18]. Photoinduced halide segregation in mixed-halide compositions is the clearest example: its onset, extent, and reversibility depend on illumination intensity and history, temperature, halide ratio, and dwell time [31]. The THz dataset compiled in this review was acquired almost entirely on as-prepared samples in the dark at room temperature and therefore characterizes the static, as-grown defect state; this is a limitation of the present evidence base rather than of the technique. Extending the fingerprint approach to operating conditions would require, in order of increasing difficulty: THz-TDS under controlled continuous illumination with defined soaking time; THz-TDS under applied bias in a transmission-compatible electrode geometry; temperature-resolved measurement across the operating window; and repeated measurement over accelerated-aging sequences. None of these is precluded by the physics of the measurement.

### 6.2. Requirements for Measurement on Complete Device Stacks

The library reported here comprises bare films and crystals. Applying THz-TDS to complete solar cells introduces several optical constraints. Transparent conductive oxides such as ITO and FTO are strongly absorbing and dispersive in the THz range because of their free-carrier response, and metal electrodes are opaque; both dominate the transmitted signal. Organic transport layers such as spiro-OMeTAD and P3HT contribute their own low-frequency vibrational features, and encapsulation glass and barrier films add thickness-dependent etalon fringes. Isolating the absorber contribution from a full stack therefore requires either reflection geometry with a carefully characterized reference, differential measurement against a stack fabricated without the absorber, or measurement at an intermediate fabrication stage before the top contact is deposited. The last of these is the most practical route for inline application and is compatible with the process-step-resolved monitoring described in Section 5.1.

### 6.3. Practical Requirements for Manufacturing Deployment

The manufacturing application proposed in Section 5 remains conceptual, and the requirements for realizing it can be stated concretely. Acquisition time: A single THz-TDS waveform with an adequate signal-to-noise ratio for LHO decomposition requires seconds to minutes with standard delay-line scanning; inline use at web speeds would require asynchronous optical sampling or electronically controlled optical sampling to reach kHz acquisition rates. Spot size and mapping: The diffraction-limited far-field spot is of order 1 mm at 1.5 THz, which sets the lateral resolution of any inline map and is coarse relative to typical defect inhomogeneity scales; near-field approaches recover resolution at the cost of throughput [23]. Thickness range: The thin-film approximation used in Section 3 holds for films that are thin compared with the THz wavelength, and the reported uncertainty figure applies to films above 100 nm, while the upper bound has not been established. Calibration transfer: Because the Ω–concentration relation is material- and process-specific, each production line and each composition would require its own calibration against XPS, with periodic re-verification. Environmental control: Water vapor absorbs strongly across the analysis window, so a purged or evacuated beam path is required. Device-stack compatibility is addressed in Section 6.2.

### 6.4. Summary Map of Defect Mechanisms, THz Signatures, and Complementary Methods

Table 4 consolidates the review by linking each defect or degradation mechanism to its expected THz signature, the supporting evidence, the confidence that can presently be placed in the assignment, and the complementary technique required for confirmation. Table 5 compiles the quantitative parameters underlying the library so that fingerprint robustness can be assessed across studies on a common basis.

The pattern visible in Table 5 is itself informative: resonance frequencies are reported consistently, whereas oscillator strengths, uncertainties, and replicate counts are reported only for MAPbI_3_. Standardized reporting of these quantities across future studies is a prerequisite for cross-laboratory comparison.

Looking ahead, critical gaps include: (i) independent replication of the defect-mode assignments by groups outside the originating laboratory; (ii) a statistically complete Ω–concentration calibration with regression statistics, replicates, and a limit of detection; (iii) resolution of the frequency discrepancy between thin-film and single-crystal *δ*/*α* FAPbI_3_ interfacial modes; (iv) a matched SVE-fabricated *γ*-CsPbI_3_ series to separate compositional from process effects; (v) time-resolved THz-TDS under pulsed illumination, bias, and elevated temperature to distinguish photo-generated from thermally stable defects; (vi) extension of the fingerprint library to ternary/quaternary compositions and 2D Ruddlesden–Popper phases; (vii) the quantitative THz oscillator strength–PCE calibration curve that would transform THz-TDS into a direct device performance predictor; and (viii) integration of THz measurement heads into inline SVE deposition systems for real-time manufacturing quality control.

## 7. Conclusions

OHP solar cells now reach 27.3% certified PCE in single-junction devices and 34.85% NLR-certified perovskite–silicon tandem values, yet grain-boundary defects remain among the principal barriers to bridging the gap with the S–Q limit. This overview has assessed the extent to which THz-TDS fulfills the role of a composition-resolved defect fingerprint tool, through a library spanning five archetypal OHP systems in both thin-film and single-crystal forms:

(1) SVE-fabricated MAPbI_3_ carries a unique 1.58 THz CH_3_NH_2_ molecular defect fingerprint (absorption coefficient > 11,000 cm^−1^) [28]. The oscillator strength scales linearly with XPS-quantified defect concentration across four annealing conditions [35], establishing a calibrated, contact-free proxy for defect concentration within that material–process combination.
(2) MAPbBr_3_ shows DFT-verified THz phonon modes at 0.8, 1.4, and 2.0 THz that are unchanged by modification of GB chemistry [36]. Under the conditions tested, no defect-induced THz mode was detected, defining the ‘clean grain boundary’ spectroscopic benchmark for bromide-based compositions.
(3) *δ*/*α*-mixed FAPbI_3_ displays phase-boundary-specific modes at 1.62 THz in SVE-fabricated flexible thin films [29] and at 2.0 and 2.2 THz at single-crystal surfaces [30]—invisible to XRD but accessible by THz—enabling non-contact phase-boundary quality assessment. The frequency difference between the two geometries is unresolved and identifies a specific target for further work.
(4) FAPb(Br,I)_3_ provides a composition-tunable THz map from defect-active (I-rich, ~1.6 THz) to defect-quiet (Br-rich, phonon-dominated), characterizing the as-grown compositional state and directly guiding halide ratio optimization [32].
(5) Solution-processed *γ*-CsPbI_3_ shows grain-size-independent bulk phonon modes at 0.9, 1.5, and 1.8 THz over grain sizes of 300–460 nm, with no detectable grain-size-dependent defect resonance [37]. Because these films were solution-processed while the MAPbI_3_ comparison films were SVE-fabricated, the result constrains rather than isolates the role of fabrication route.

Looking forward, the establishment of a direct ‘THz Fingerprint–to–PCE’ calibration curve will be the final step in transitioning THz-TDS from a laboratory diagnostic tool to a predictive manufacturing engine [41]. By integrating THz measurement heads directly into inline SVE deposition systems, real-time feedback loops can be realized to optimize passivation strategies without the need for full device fabrication. This shift toward spectroscopy-guided manufacturing offers a scalable and cost-effective pathway to close the remaining efficiency gap with the S–Q limit, ensuring both the high performance and long-term reliability required for the commercialization of perovskite photovoltaics. Realizing it will require independent replication, statistically complete calibration, measurement under operating conditions, and the device-stack and throughput requirements set out in Section 6.

## Figures and Tables

**Table 2:** The three-pillar THz defect engineering framework: capabilities, evidence base, status, and application scope.

Pillar	Core Capability	Key Evidence	Status	Application
I	THz oscillator strength Ω (1.6 THz) as contact-free defect proxy (<5% uncertainty)	Linear Ω—[CH_3_NH_2_] correlation in MAPbI_3_ (samples A–D) [35]	Demonstrated for MAPbI_3_/SVE; regression statistics, replicates and detection limit not yet established	Substrate-by-substrate process monitoring; inline SVE QC without device fabrication
II	Material-specific THz fingerprint database spanning 5 OHP systems	Defect-active (MAPbI_3_) [28]; defect-quiet (MAPbBr_3_) [36]; phase-sensitive (FAPbI_3_) [29,30]; tunable (FAPb(Br,I)_3_) [32]; inorganic-clean (*γ*-CsPbI_3_) [37]	Demonstrated; independent replication outstanding for all defect-mode assignments	Non-contact discrimination of composition, phase, and halide ratio in a single scan
III(a)	THz-verified defect elimination	N_2_ annealing (110 °C/45 min): Ω → 0 [35]	Demonstrated	Iterative annealing optimization without full device fabrication
III(b)	THz-guided verification of chemical and interfacial passivation	E-bias: 55% CH_3_NH_2_ eliminated (XPS) [38]; P3HT HTL: 2-week stability [39]; sequential ligands: >93% PCE retention [14]	Proposed; no before/after THz data reported	Prospective spectroscopic certification of passivation

**Table 3:** Comparative capabilities of THz-TDS vs. conventional OHP defect characterization methods for standard laboratory implementations. Y = capable; N = not capable; P = partial or implementation-dependent.

Criterion	THz-TDS	XPS	PL	Impedance	XRD/SEM
Defect species ID	P (indirect, via calibration)	Y Direct	N Indirect	N Indirect	N None
Non-contact	Y	N UHV	Y	N Contacts	Y
Non-destructive	Y	P	Y	P	Y
Quantitative	P (calibrated proxy)	P	P	P	N
Ambient/RT	Y	N	Y	P	Y
Full-depth probe	Y	N ~10 nm	Y	Y	Y
Phase sensitivity	Y Interfacial	N	P	N	Y Bulk

**Table 4:** Map linking defect and degradation mechanisms to THz signatures, supporting evidence, confidence level, and complementary characterization.

Mechanism	Expected THz Signature	Supporting Evidence	Confidence	Complementary Method
CH_3_NH_2_ molecular defect at GB (SVE MAPbI_3_)	Additional narrow mode at 1.58–1.62 THz; Ω scales with concentration	Process dependence; XPS correlation; DFT mismatch for intrinsic modes [28,35]	High for detection; moderate for absolute quantification	XPS for chemical identity and calibration
Halide vacancies and interstitials	None expected (THz-silent; negligible lattice distortion)	Absence of assignable modes in any composition reviewed	Low; effectively undetectable by far-field THz-TDS	DLTS, impedance spectroscopy, first-principles defect calculations [17,25,26]
*δ*/*α* phase boundary (FAPbI_3_)	1.62 THz (thin film) or 2.0 and 2.2 THz (single crystal)	Appears only in mixed phase; MD assignment; invisible to XRD [29,30]	Moderate; frequency not transferable between geometries	XRD for phase fraction; XPS for FA chemical state
Halide compositional non-uniformity (FAPb(Br,I)_3_)	~1.6 THz absorption in I-rich compositions	Composition series across *x* [32]	Moderate; as-grown state only	PL peak-shift mapping; XRD; illumination-resolved measurement
Photoinduced halide segregation under operation	Predicted time-dependent growth of the I-rich feature	Not yet measured by THz	Proposed only	*In situ* PL and XRD under illumination [18,31]
GB structural disorder without molecular residue	No additional mode; intrinsic phonons only	*γ*-CsPbI_3_ grain-size series [37]	Moderate; bounded by tested grain-size range and by solution processing	SEM/AFM for grain statistics; XPS for chemical state
Aging and decomposition to PbI_2_	Monotonic decay of the 0.968 THz absorption	Real-time aging measurement [42]	Moderate; single study	XRD for PbI_2_ formation; UV–vis
Bulk, interfacial, and contact-related losses	Not accessible in the film-only geometry used here	Outside the scope of the present dataset	Not applicable	Device-level impedance, transient photovoltage, *operando* methods [17,18]

**Table 5:** Cross-study quantitative summary of the reported THz fingerprints.

System/Mode	Frequency (THz)	Oscillator Strength/Absorption Metric	Uncertainty	Thickness	Substrate	Processing	Samples	Independent Validation
MAPbI_3_ defect mode	1.58 (2019); 1.62 (2020)	*α* > 11,000 cm^−1^; ~50% suppression; Ω ~ 0.37 (sample A)	<5% repeat-fit, films > 100 nm	~300 nm	Al_2_O_3_	SVE	4 anneal conditions	None reported
MAPbI_3_ intrinsic modes	0.95/1.87 (2019); 0.97/2.01 (2020)	Constant across anneal conditions	Not reported	~300 nm	Al_2_O_3_	SVE	4	Consistent with [20,21]
MAPbBr_3_ phonons	0.8, 1.4, 2.0	Unchanged before/after 150 °C anneal	Not reported	Not stated	Al_2_O_3_	SVE	2 conditions	Consistent with [21,22]
FAPbI_3_ mixed phase (film)	1.62	~40% absorptance	Not reported	91 nm PbI_2_ + 209 nm FAI	ITO/PEN (flexible)	SVE, 140 °C/10 min	5 anneal times	None reported
FAPbI_3_ interfacial (crystal)	2.0 and 2.2	Not reported quantitatively	Not reported	Bulk crystal	n/a	140 °C/30 min	3 phase states	None reported
FAPb(Br_x_I_1−x_)_3_	~1.6 (I-rich)	Composition-dependent	Not reported	Not stated	Al_2_O_3_	SVE	Composition series	None reported
*γ*-CsPbI_3_	0.9, 1.5, 1.8	Real conductivity 10–40 S cm^−1^ (0.5–3.0 THz)	Not reported	~300 nm	Al_2_O_3_	Solution	3 grain sizes	None reported

## Data Availability

No new data were created or analyzed in this study.
